# Solitary Lytic Bone Metastasis: A Rare Presentation of Small Lymphocytic Leukemia

**DOI:** 10.1155/2018/6154709

**Published:** 2018-10-30

**Authors:** Heather Katz, Steven Sagun, Doreen Griswold, Mohamed Alsharedi

**Affiliations:** ^1^Department of Hematology/Oncology, Joan C. Edwards School of Medicine Marshall University, 1600 Medical Center Dr, Huntington, WV 25701, USA; ^2^Joan C. Edwards School of Medicine Marshall University, 1600 Medical Center Dr, Huntington, WV 25701, USA; ^3^Department of Pathology, Joan C. Edwards School of Medicine Marshall University, 1600 Medical Center Dr, Huntington, WV 25701, USA

## Abstract

Chronic lymphocytic leukemia (CLL)/small lymphocytic lymphoma (SLL) is a hematologic malignancy characterized by an over accumulation of incompetent neoplastic lymphocytes. Bone metastasis in CLL/SLL is very rare. We report a case of a 76-year-old Caucasian female presented with an unresolving pulmonary infiltrate with mediastinal lymphadenopathy concerning for malignancy. Positron emission tomography (PET)/computed tomography (CT) showed an infiltrative mass in the mediastinum with diffuse uptake and a hypermetabolic mass within the left iliac bone. Transbronchial biopsy revealed morphology and features of SLL. However, with concern for another primary cancer, a CT-guided biopsy of the PET avid left iliac bone was performed and revealed bone and marrow with involvement of CLL/SLL similar to the chest lymphadenopathy. To our knowledge and after extensive review of medical literature, this is first reported case of SLL with solitary bone metastasis to the hip.

## 1. Introduction

Chronic lymphocytic leukemia (CLL)/small lymphocytic lymphoma (SLL) is an insidious malignancy in which the bone marrow produces too many lymphocytes thus accruing neoplastic lymphocytes within the lymph nodes, blood, or bone marrow [1, 2]. CLL/SLL is currently the most common leukemia in the USA and will account for approximately one-third of all adult leukemia incidences in 2018 [1–3]. Identification of ≥5000 lymphocytes per *μ*L with proper immunophenotype is diagnostic CLL while SLL requires lymphadenopathy and/or splenomegaly with <5000 lymphocytes per *μ*L in the peripheral blood [2, 4, 5]. Bone metastasis in CLL/SLL is very rare, generally osteolytic, and affects less than 5% of CLL patients [6–9]. We report a very rare case of a 76-year-old female who was incidentally found to have diffuse lymphadenopathy on imaging, subsequently diagnosed with CLL/SLL and found to have a solitary left iliac bone lesion consistent with CLL/SLL.

## 2. Case Report

A 76-year-old Caucasian female with a past medical history of chronic obstructive pulmonary disease (COPD), hypertension, and osteoarthritis had developed a COPD exacerbation requiring steroids and antibiotics. Her primary care physician ordered a chest X-ray (CXR) due to persistent cough and abnormal breath sounds on physical exam. The CXR revealed a moderate-sized infiltrate in the inferior portion of lingular segment which likely represented a pneumonic infiltrate. She was treated with a 10-day course of antibiotics. Repeat chest X-ray revealed minimal clearing of parenchymal infiltrate from the lingular segment. A subsequent computed tomography (CT) scan of the chest showed evidence of residual infiltrative changes involving the right middle lobe as well as the lingular division of the left upper lobe. There was also evidence of diffuse low-attenuation density involving the mediastinum highly suggestive of diffuse adenopathy which was concerning for lymphoma.

**Figure 1:**
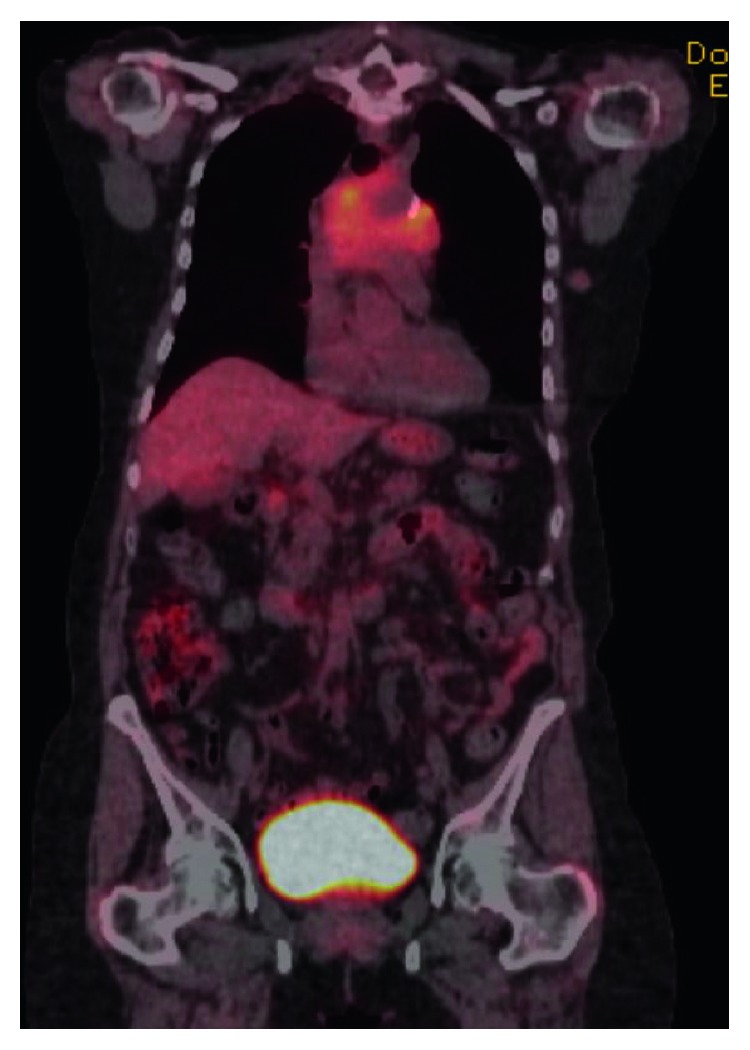
PET/CT with an infiltrative mass in the mediastinum with diffuse uptake.

At initial consultation by oncology, her vital signs were stable, and she denied B symptoms including fevers, night sweats, and weight loss. She denied any hemoptysis or worsening shortness of breath. Physical exam was unremarkable with no palpable cervical, axillary, or inguinal adenopathy or hepatosplenomegaly, and respiratory exam was clear to auscultation bilaterally. CBC with differential showed a white blood cell count of 4.4 × 10 mm^3^ with an absolute lymphocyte count of 0.66 × 10 mm^3^, hemoglobin of 13.7 gm/dL, and platelet count of 178 × 10 mm^3^. She had an unremarkable complete metabolic panel (CMP) and mildly elevated lactate dehydrogenase (LDH) at 235. Due to concern for lymphoma and findings on CT of the chest, a PET/CT was ordered which showed an infiltrative mass in the mediastinum with diffuse uptake (maximum standardized uptake value (SUV) 5.94 (Figure 1).

**Figure 2:**
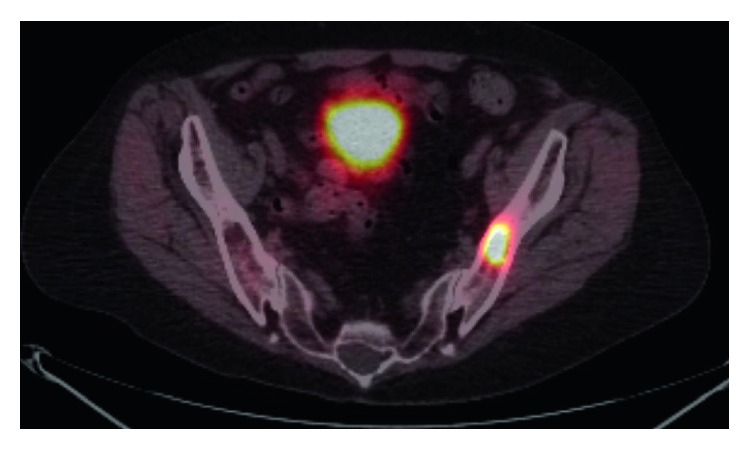
PET CT with hypermetabolic mass within the left iliac bone.

There were small lymph nodes in the left axilla showing low level uptake with maximum SUV 1.73 and 1.52, respectively. Finally, there was a hypermetabolic mass within the left iliac bone with a maximum SUV 11.71 (Figure 2).

**Figure 3:**
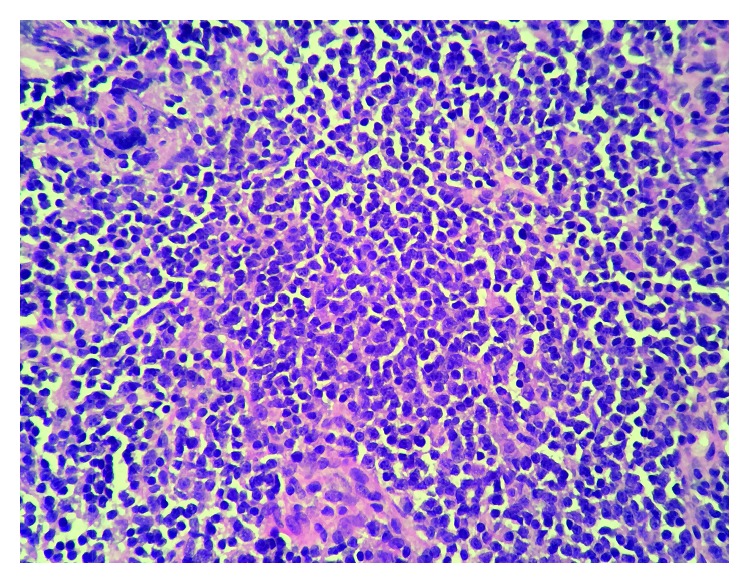
Hematoxylin and eosin stain: lymphoid tissue composed of small, mitotically inactive cells with round to slightly irregular nuclear contours and scant cytoplasm.

She underwent an endobronchial ultrasound and transbronchial biopsy of station 7 lymph node which revealed lymphoid tissue composed of small, mitotically inactive cells with round to slightly irregular nuclear contours and scant cytoplasm (Figure 3).

**Figure 4:**
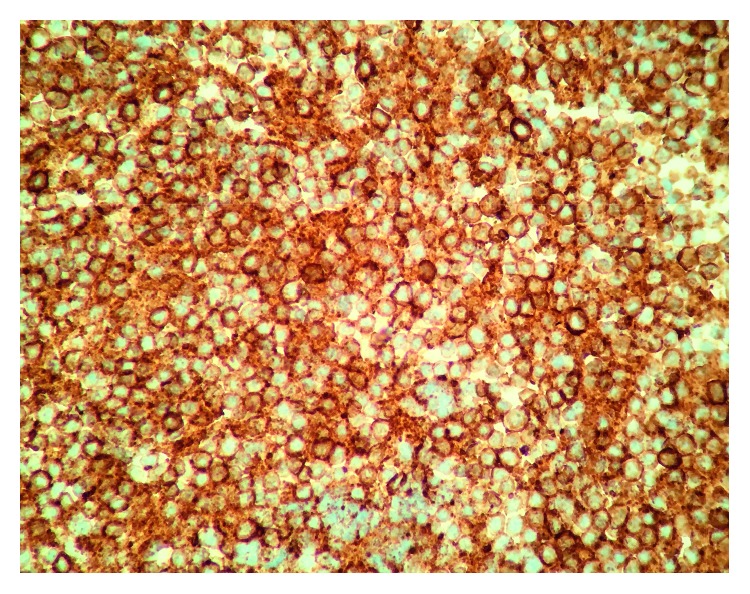
Immunohistochemistry positive for CD20.

**Figure 5:**
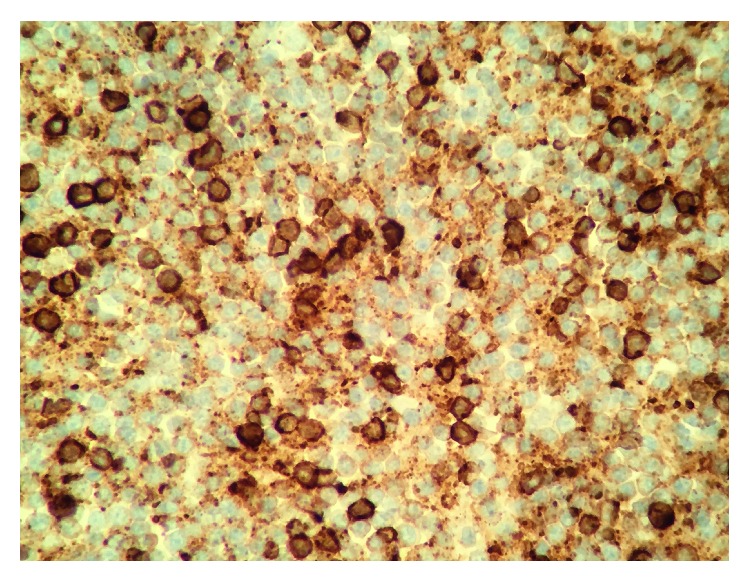
Immunohistochemistry positive for CD5.

**Figure 6:**
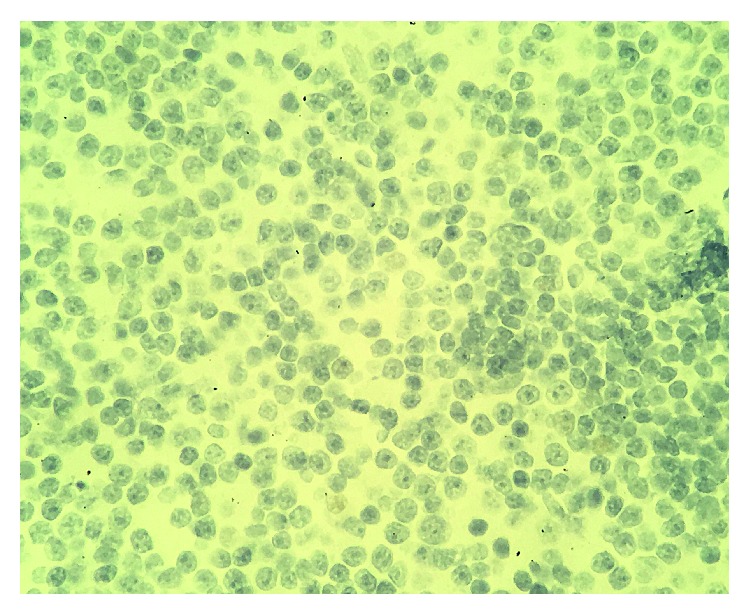
Immunohistochemistry negative for Cyclin D1.

Flow cytometric analysis demonstrated an abnormal CD5+ B cell population. Immunohistochemical stains showed that the cells were positive for CD20 (Figure 4) and CD5 (Figure 5) and negative for Cyclin D1 (Figure 6).

**Figure 7:**
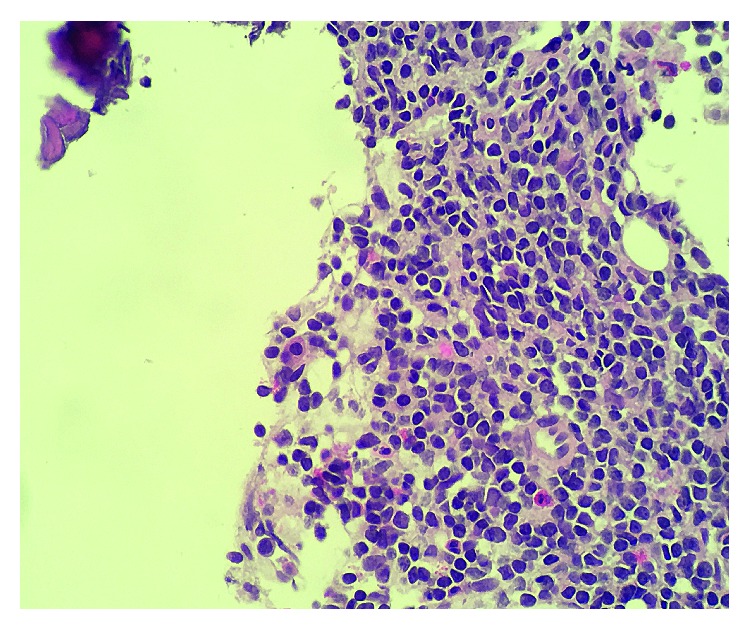
Hematoxylin and eosin stain: a malignant infiltrate of small, mitotically inactive lymphocytes replacing the marrow space in the biopsy from left iliac bone.

Scattered CD3 positive T cells were also present. The morphology and phenotype supported the diagnosis of small lymphocytic leukemia (SLL). Since SLL does not typically present with bone lesions and there was concern for another primary cancer, a CT-guided biopsy was performed of the PET avid left iliac bone. Pathology from that biopsy showed both bone and marrow with involvement of CLL/SLL (Figure 7).

**Figure 8:**
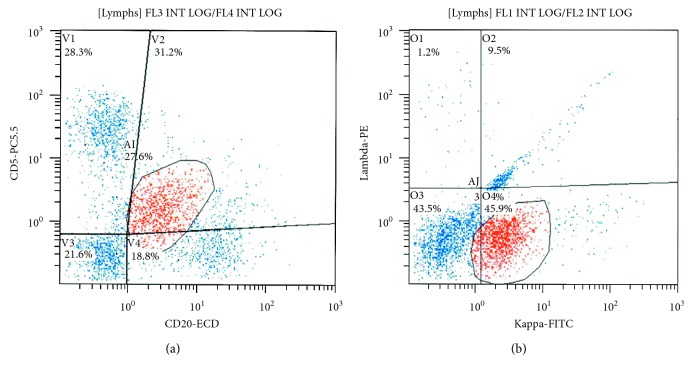
Flow cytometry from the left iliac bone biopsy revealed monoclonal kappa light chain restricted B-cell population phenotypically consistent with CLL/SLL.

Flow cytometry from the left iliac bone biopsy revealed monoclonal kappa light chain restricted B-cell population phenotypically consistent with CLL/SLL (Figure 8).

No specific abnormalities were detected by CLL fluorescence in situ hybridization (FISH) including centromere 12, 13q14 (DLEU1), ATM/11q, TP53/17p13, and CCNDQ/IGH–t(11; 14).

Currently, she does not have cytopenias, B symptoms, or bulky disease; however, there was concern that the mediastinal adenopathy may be contributing to her pulmonary symptoms and that the left hip lesion was causing discomfort. Consequently, systemic therapy was offered as was radiation to the hip; however, the patient declined and opted for observation and close surveillance. She will return for further evaluation of symptoms and laboratory data in 2 months.

## 3. Discussion

CLL/SLL is a chronic disease characterized by an over accumulation of small dysfunctional neoplastic lymphocytes. Hypothesized pathogenesis of the dysfunctional lymphocytes includes environmental factors, chromosomal deletions, genetic or epigenetic modifications, and altered miRNA expression [1, 2]. The resulting lymphoproliferation causes harm to the patient due to suboptimally functioning B-cells and overcrowding of the blood, bone marrow, lymph nodes [2].

CLL/SLL is currently the most common leukemia in the USA and will account for approximately one-third of all adult leukemia incidence in 2018 [1–3]. The median age at diagnosis is 70 years, and there is a 1.7 : 1 male: female ratio [1, 4]. The symptoms and signs of CLL/SLL are variable. Although many patients are asymptomatic at time of diagnosis, late-stage CLL/SLL can present with B symptoms (fever, night sweats, and weight loss), lymphadenopathy, hepatosplenomegaly, or skin lesions. The most common laboratory abnormalities are lymphocytosis, anemia, and thrombocytopenia, or hypogammaglobulinemia [2, 10].

The workup for CLL/SLL includes a complete blood count (CBC) with differential, peripheral smear evaluation, and flow cytometry of the atypical lymphocytes. Identification of ≥5000 B-cells per *μ*L with proper immunophenotype is diagnostic of CLL while SLL requires lymphadenopathy and/or splenomegaly with <5000 B-cells per *μ*L in the peripheral blood with proper immunophenotype [2, 4, 5]. The typical immunophenotype of CLL is CD5 positive, CD23 positive, CD10 negative, CD19 positive, CD20 dim, surface immunoglobulin dim positive, and cyclin D1 negative [2, 4, 5].

While CLL is the most common leukemia, CLL associated solitary lytic bone lesions are very uncommon and affects less than 5% of CLL patients [6–9]. Secondary nonlymphoid malignancies should also be considered, and large retrospective cohort studies have shown increased relative risk and prevalence of lung cancer in CLL patients [11].

Bone lesions associated with CLL are generally osteolytic, and the pathogenesis of bone osteolysis is thought to be caused by localized osteoclast stimulating factors and cytokines released by the abnormal lymphocytes [1, 6, 9, 12, 13]. Narayan et al. suggests that CLL bone lesions are most common in the axial skeleton and proximal long bones [6]. A review of 14 case reports supports this hypothesis as 87% of the reviewed cases were CLL bone lesions involving the axial skeleton or proximal long bones [6–9, 12–16]. The most common site of metastasis was to the femur and vertebral column [6–9, 12–16]. In 70% of the reports, patients had multiple bone lesion sites [6–9, 12–16]. After extensive review of the literature, solitary lytic bone lesions of the hip from SLL/CLL were not reported.

## 4. Conclusion

Solitary bone lesions due to chronic lymphocytic leukemia are extremely rare, and this is the first case presenting as a solitary hip lesion. This case highlights the importance of a multidisciplinary and comprehensive workup in patients who present with mediastinal lymphadenopathy and solitary bone lesions. We sought to highlight this case for its rarity and unusual presentation and to add further information in the literature about atypical metastatic involvement in SLL/CLL.
